# Mind After Uexküll: A Foray Into the Worlds of Ecological Psychologists and Enactivists

**DOI:** 10.3389/fpsyg.2020.00480

**Published:** 2020-03-24

**Authors:** Tim Elmo Feiten

**Affiliations:** Department of Philosophy, University of Cincinnati, Cincinnati, OH, United States

**Keywords:** enactivism, ecological psychology, *Umwelt*, Jakob von Uexküll, embodied cognition, philosophy of nature

## Abstract

For several decades, a diverse set of approaches to embedded, embodied, extended, enactive and affective cognition has been challenging the cognitivist orthodoxy. Recently, the prospect of a combination of ecological psychology and enactivism has emerged as a promising candidate for a single unified framework that could rival the established cognitivist paradigm as “a working metatheory for the study of minds” (Baggs and Chemero, 2018, p. 11). One obstacle to such an ecological-enactive approach is the conceptual tension between the firm commitment to realism of those following James Gibson’s ecological approach and the central tenet of enactivism that each living organism enacts its own world, interpreted as a constructivist or subjectivist position. Baggs and Chemero (2018) forward the concept of *Umwelt*, coined by the biologist Jakob von Uexküll, as a conceptual bridge between the two approaches. Inspired by Kant, Uexküll’s *Umwelt* describes how the physiology of an organism’s sensory apparatus shapes its active experience of the environment. Baggs and Chemero use this link between the subject and its objective surroundings to argue for a strong compatibility between ecological psychology and enactivism. Fultot and Turvey on the other hand view *Umwelt* as steeped in representationalism, the rejection of which is a fundamental commitment of radical embodied cognition (Fultot and Turvey, 2019). Instead, they advance Uexküll’s “compositional theory of nature” as a conceptual supplement for Gibson’s ecological approach (von Uexküll, 2010, p. 171; Fultot and Turvey, 2019). In this paper, I provide a brief overview of Uexküll’s thought and distinguish a crucial difference between two ways of using his term *Umwelt*. I argue that only one of these ways, the one which emphasizes the role of subjective experience, is adequate to Uexküll’s philosophical project. I demonstrate how the two ways of using *Umwelt* are employed in the philosophy of cognitive science, show how this distinction matters to recent debates about an ecological-enactive approach, and provide some critical background to Uexküll’s compositional theory of meaning.

## Introduction

For several decades, a set of diverse but related approaches has been challenging the cognitivist orthodoxy. By recognizing the mind as embedded, embodied, extended, enactive, and affective (4EA), they have invigorated debates in the philosophy of cognitive science on a wide number of topics. Their diversity of methods and concerns is both a strength and a weakness: many of the different tendencies within 4EA cognition draw on disparate conceptual sources and so far, the various perspectives have not coalesced into a single unified framework that could rival the established cognitivist paradigm as “a working metatheory for the study of minds” (Baggs and Chemero, 2018, p. 11). Recently, attempts have gained traction to create such a common framework through a combination of ecological psychology and enactivism. For such an ecological-enactive approach to emerge, the conceptual foundations of its two components have to be in harmony with each other.

One of the main sources of tension between ecological psychology and enactivism is the contrast between the firm commitment to realism of those following James Gibson’s ecological approach and the central tenet of enactivism that each living organism enacts its own world, interpreted as a constructivist or subjectivist position. Baggs and Chemero forward the concept of *Umwelt*, coined by the biologist Jakob von Uexküll, as a conceptual bridge between the two approaches (Baggs and Chemero, 2018). Inspired by Kant, Uexküll’s *Umwelt* describes how the physiology of an organism’s sensory apparatus shapes its active experience of the environment. Baggs and Chemero use this link between the subject and its objective surroundings to argue for a strong compatibility between ecological psychology and enactivism. Fultot and Turvey on the other hand view *Umwelt* as steeped in representationalism, the rejection of which is a fundamental commitment of radical embodied cognition (Fultot and Turvey, 2019). Instead, they advance Uexküll’s “compositional theory of nature” as an account of how meaning takes place in the interactions between biological entities that meshes better with Gibson’s ecological approach (von Uexküll, 2010, p. 171; Fultot and Turvey, 2019). However, the roots of Uexküll’s musical theory of meaning in neovitalism and romantic holism have to be considered carefully before we can evaluate the costs and benefits of potentially importing it into the philosophy of embodied cognition.

There is still a lot of work to be done in piecing the parts together for a united ecological-enactive paradigm for cognitive science. If Uexküll’s thought is to provide one or more pieces of this puzzle, the necessary first step is to give a clear picture of what those pieces are, how they might connect to the philosophy of embodied cognition, and what philosophical commitments come with each of them. To this end, I provide a brief overview of Uexküll’s thought and distinguish a crucial difference between two ways of using his term *Umwelt*. I argue that only one of these ways, the one which emphasizes the role of subjective experience, captures Uexküll’s philosophical impetus. Simultaneously, it is only this second interpretation of *Umwelt* that really connects to the source of the tension between ecological psychology and enactivism. I will then use some examples from the literature to demonstrate how the two ways of using Umwelt are employed in the philosophy of cognitive science, show how this distinction plays out in recent debates, and provide some critical background to Uexküll’s compositional theory of meaning^1^.

## The Many Worlds of Jakob von Uexküll

Jakob von Uexküll was an Estonian-born biologist who is considered a predecessor of cybernetics, a pioneer of ethology and even as the “founder of two separate disciplines, ecology and semiotics” (Amrine, 2015, p. 47). Born in 1864, he published his most influential works between 1909 (*Umwelt und Innenwelt der Tiere*) and 1940 (*Bedeutungslehre*, translated as *A Theory of Meaning*). A physiologist by trade, Uexküll conducted extensive experimental studies prior to his career as a writer. Beginning in 1891 with experiments on the nervous systems of frogs conducted in Heidelberg, Uexküll soon moved on to the sea creatures that would become his specialty, studying first squid in Naples and later sea urchins on the coast of the Indian Ocean at Daressalam until 1900 (Mildenberger and Herrmann, 2014b, pp. 274–279). At the beginning of this decade of empirical work, Uexküll was still convinced that biological phenomena could be explained by mechanistic principles.

Throughout his research, he encountered increasing difficulties to account for the phenomena he observed by purely mechanistic means and came to endorse a philosophy of nature influenced by neovitalism and romanticist holism. These developments culminated in his view that the “machine theory of living beings” is fundamentally flawed (von Uexküll, 2010, p. 41). The “machine theorists” hold that “all living things are only machines” and treat them as “pure objects” (von Uexküll, 2010, pp. 41, 42). Instead, biology can only understand the nature of organisms by treating them as subjects experiencing and inhabiting their own worlds (von Uexküll, 2010, p. 41). Uexküll advanced a model of how perception and action link the organism’s nervous system with its environment in a *functional cycle*: “everything a subject perceives belongs to its perception world [*Merkwelt*], and everything it produces, to its effect world [*Wirkwelt*]. These two worlds, of perception and production of effects, form one closed unit, the *environment* [*Umwelt*]” (von Uexküll, 2010, p. 42).

In its full sense, the *Umwelt* refers to the phenomenal world which an individual organism constructs for itself by turning physical stimuli into patterns of neuronal excitation which constitute signs. The *Umwelt* constitutes the sum total of the subject’s experience, but the process in which the organism constructs its own *Umwelt* is not conscious and not accessible to the subject in its experience. Instead, the meaningful objects and the space in which we encounter them appear to us as objective reality. This reading captures Uexküll’s central concern for the animal as subject, in an explicitly Kantian^2^ sense:

“The task of biology consists in expanding in two directions the results of Kant’s investigations:—(1) by considering the part played by our body, and especially by our sense-organs and central nervous system, and (2) by studying the relations of other subjects (animals) to objects” (von Uexküll, 1926, p. 15).

I will refer to this full sense of *Umwelt* as the world of subjective experience constructed by the organism itself as type 2 *Umwelt*.

In a deflated reading, *Umwelt* is often understood merely as that subset of physical properties which are accessible to the perception and action of an organism based on its physiology. The entire aspect of subjective experience is lost here, and the specific physiological makeup of a single organism is still fully within the scope of a purely external, quantitative description. This sense of *Umwelt* I call type 1 *Umwelt*. Uexküll himself sometimes uses type 1 *Umwelt* and speaks about *Umwelt* as if it resulted from a mere selection, as a “tiny excerpt,” “small section,” or “only a piece cut out of its surroundings” (von Uexküll, 2010, pp. 53, 133). Most of the time he uses type 2 *Umwelt*, which lies at the heart of his entire intellectual project. Unfortunately, the majority of uses of the term *Umwelt* in the literature since Uexküll use type 1 *Umwelt* (Mildenberger and Herrmann, 2014b, pp. 264, 265).

The reasons for adopting type 1 *Umwelt* rather than type 2 can be traced to two main differences in interpretation: First, *Umwelt* is sometimes described as the result of a mere selection, rather than the more intricate process of construction. But the material world as described by physicists contains no experience, and in order to get from physical perturbations to experience, the subject has to construct the *Umwelt*. Experience is not just a set of carefully selected physical perturbations. Second, the specific structure of an *Umwelt* is sometimes described at the level of a species, rather than an individual organism. This makes sense insofar as all bees have very similar sensory organs, and the structure of their experience is likely to be very similar, while being very different from the structure of human experience. However, a species does not, as far as we know, have experience—individual organisms do. If *Umwelt* is the world as experienced by an organism, this is not an abstract model organism standing in for a whole species, but a concrete individual living being. While there are many situations in which it makes sense to talk about the kind of environment described by type 1 *Umwelt*, it excludes the majority of philosophical points that are central to Uexküll’s thought. Some examples will help illustrate this point.

Mildenberger and Herrmann (2014a, p. 10) consider Uexküll to be a predecessor of the concept of niche construction, which they regard as a “more poignant version of the Umwelt concept.”. The link is plausible. Uexküll’s description of *Echinocardium caudatum* burrowing into the sand through the wavelike motion of countless tiny bristles with spoon-shaped, widened points is a particularly apt illustration of niche construction. However, the notion of construction that is central to Uexküll’s philosophy, and that will be used in this paper, refers only indirectly to the shaping of the physical environment by the organism. The construction of the Umwelt is the generation by the organism of the world it experiences^3^. Uexküll explicitly models this process on Kant’s account^4^ of how the transcendental subjectivity provides the necessary structure that makes our experience possible: “with Kant, we make the constructive activity of the subject the very center of our consideration” and understand “space as the means whereby we construct external experience” (von Uexküll, 1926, p. 19). Brentari (2013, p. 17) summarizes the “transcendental construction of the Umwelt”: “the stimuli coming from the outside reality are translated into signs by the nervous system, then the physiologically produced signs are transposed outwards and, finally, they are experienced as objective qualities of the world.” Only by locating all the signs it has constructed outside of itself does the subject span open the spatial dimensions of its own experiential world, somewhat like opening an umbrella. This world that is experienced by the organism which has constructed it is at the heart of Uexküll’s thought. I will refer to it as type 2 *Umwelt* in contrast to the deflationary usage of type 1 *Umwelt*.

Many of the examples that Uexküll uses reappear throughout his works, but their presentation is often subtly different from text to text. The case of the semicircular canals helps illustrate how Uexküll expresses the same idea in different ways that emphasize either type 1 or type 2 *Umwelt*. In *Forays*, Uexküll describes the effect space of humans and relates its three-dimensional structure to the semi-circular canals in the inner ear (von Uexküll, 2010, pp. 54–56). We can understand the relationship between effect space and the physiology of the inner ear purely in terms of type 1 *Umwelt:* The observable behavior of the experimental subject and their verbal reports allow us to investigate the space that structures their behavior, while the physiology of the semi-circular canals provides a potential mechanism in the organism that can ground the capabilities of the subject to interact with their environment in a way that is spatially structured. The entire connection between spatial behavior and the inner ear can be explained without any special reference to the subjective experience of the organism.

The same connection is described in *Bausteine zu einer biologischen* Weltanschauung (1913) with much the same content but very different implications. As in *Forays*, Uexküll compares the semi-circular canals to a coordinate system with three dimensions that allows us to experience objects as located in space. In this early text, however, the role of the semi-circular canals is explicitly introduced as an update to the Kantian account of the three spatial dimensions. In Uexküll’s account, Kant declared space to be a “structural element of our soul” which exists before any external impressions take place and which allows them to be synthesized into unities (von Uexküll, 1913, pp. 284–286). Uexküll considers Kant’s claim that the three dimensions of space exist preformed in our “soul” without the need for any external cause to be outdated: the discovery of the role played by the semi-circular canals has provided us with a physiological substitute for Kant’s idealist subjectivity (von Uexküll, 1913, pp. 286, 287). This is a rare critical note, as Uexküll presents his thought as a more harmonious continuation of Kant’s enterprise in his later texts (von Uexküll, 1926, p. 15). It is worth pointing out this earlier stance as a counterweight to the view of Uexküll as naively overestimating his proximity to Kant (Langthaler, 1992, pp. 232–234; Winthrop-Young, 2010, pp. 230, 231). Even though Uexküll’s reading of Kant may be unsophisticated and philosophically crude, he was aware of the crucial difference between Kant’s teachings and his own empirically informed account of the constructive process that gives rise to *Umwelt*.

Part of the difficulty with Uexküll’s concept of *Umwelt* arises because at different times he writes about the environments of animals as a scientist or as a philosopher of nature, and sometimes as both. When Uexküll invites us to “make a bubble around each of the animals living in the meadow” and imagine these bubbles as their *Umwelten*, he invents for us an exercise of the philosophical imagination (von Uexküll, 2010, p. 43). The poetic tone of the *Forays* is not due to an arbitrary stylistic preference. Uexküll studied Kant together with the poet Rainer Maria Rilke, and his respect for nature was a deeply personal attitude (Winthrop-Young, 2010, pp. 230, 231). But the reason for this change in style is more systematic: When the description moves from functional cycles to bubbles, Uexküll the scientist has passed the baton to Uexküll the philosopher of nature. There are two very different intellectual activities involved here: a scientific research program into the behavior of animals and a philosophy of nature that considers each organism as a subject experiencing its own world (Godfrey-Smith, 2001). While the two are deeply connected, there are also clear differences in their methods and limits, and depending on the task at hand, Uexküll moves freely between the two perspectives. These shifts are not made explicit, which makes them hard to track. The transition from type 1 to type 2 *Umwelt* is the move from a scientific research project to a philosophy of nature. It is clear that in the case of the bubbles we are dealing with experience: in imagining ourselves entering the bubble and seeing the world transformed, we are imagining the experience of the animal.

The role of experience is absolutely crucial for the concept of *Umwelt*, which is why any account of it that omits type 2 *Umwelt* is problematic. *Umwelt* as the world experienced by a living subject is at the heart of Uexküll’s project. It grounds his most important points, from his insistence that every organism is a living subject and not just a machine all the way to his claim that the limitations posed to the knowledge of any subject by its Umwelt apply also to human scientists, which concludes both *Foray* and *A Theory of Meaning:*

“We can certainly get closer to all things through the use of increasingly precise apparatuses, but we do not gain any more sensory organs thereby, and all the properties of things, even when we analyze them down to the smallest details—atoms and electrons—will always remain only perception marks of our senses and ideas” (von Uexküll, 2010, p. 207).

The situation that Uexküll describes is the same one that Varela, Thompson, and Rosch diagnose at the start of *The Embodied Mind*: “we are in a world that seems to be there before reflection begins, but that world is not separate from us” (Varela et al., 1991, p. 3). Uexküll shares with enactivism the awareness that philosophers and scientists investigating minds should never lose sight of their own minds as the context in which these investigations take place. Similarly, they both start from the conviction that mind cannot be explained while ignoring experience. This is why any productive reception of Uexküll’s thought in the philosophy of cognitive science will have to grapple with type 2 *Umwelt*, and it is also why doing so might be relevant for the task of furthering rapport between ecological psychology and enactivism.

## *Umwelt* in the Philosophy of Cognitive Science

Discussions of Uexküll in the philosophy of cognitive science have become more detailed recently, especially in debates about embodied cognition. Yet a deeper sensibility for his thought has only just begun to spread, and several decades of Uexküll’s presence in the literature as a mere foot- or sidenote have shaped the vague and sometimes distorted image of his thought that is now still prevalent. It is worth briefly illustrating the range of these different interpretations or misreadings of Uexküll’s thought. Members of the general philosophical audience interested in the study of cognition might well encounter Uexküll for the first time in the works of Daniel Dennett. In his 2015 he writes:

“Every organism, whether a bacterium or a member of Homo sapiens, has a set of things in the world that matter to it and which it (therefore) needs to discriminate and anticipate as best it can. Call this the ontology of the organism, or the organism’s ‘Umwelt’ (von Uexküll, 1957). This does not yet have anything to do with consciousness but is rather an ‘engineering’ concept, like the ontology of a bank of elevators in a skyscraper: all the kinds of things and situations the elevators need to distinguish and deal with. An animal’s ‘Umwelt’ consists in the first place of affordances (Gibson, 1979), things to eat or mate with, openings to walk through or look out of, holes to hide in, things to stand on, and so forth. We may suppose that the ‘Umwelt’ of a starfish or worm or daisy is more like the ontology of the elevator than like our manifest image. What’s the difference?” (Dennett, 2015, pp. 11, 12).

The difference is that organisms are living subjects and machines are not. It is immediately clear that Dennett’s use of the term *Umwelt* is diametrically opposed to Uexküll’s in a crucial dimension: For Dennett, there is absolutely no difference between living organisms and machines, while for Uexküll this difference is of primary importance, and the whole project of developing a philosophically grounded concept of Umwelt is launched as a direct attack on the “machine theory of living beings” (von Uexküll, 2010, p. 41). What exactly *Umwelt* has to do with consciousness depends on the precise definition in play, but *Umwelt* is directly related to subjective experience. In the same text, Dennett rehearses his position that there is no “no double transduction in the brain” that would transduce the neuronal spike trains into “qualia, conceived of as states of” “the medium of consciousness” (Dennett, 2015, p. 11). It is precisely this position which Dennett rejects that meshes rather well with Uexküll’s account of *Umwelt*. The process whereby “the stimuli coming from the outside reality are translated into signs by the nervous system” which “are transposed outwards and […] experienced as objective qualities of the world” can be understood as the construction of just this medium of consciousness that Dennett denies (Brentari, 2013, p. 17). Dennett’s account of *Umwelt* is opposed to Uexküll’s in at least two respects: the relationship between experience and the brain, and the relationship between animals and machines. It is of course perfectly legitimate for Dennett to disagree with Uexküll, but giving the appearance of agreement where none exists is bound to mislead. Since his most recent book *From Bacteria to* Bach and Back (2017) contains the same account of *Umwelt* as the passage cited above, it is possible that many members of Dennett’s (2017) large audience will be first introduced to a distorted version of Uexküll’s thought.

Within the field of embodied cognition, one of the most widely read texts in which readers may encounter a brief description of Uexküll’s *Umwelt* is Andy Clark’s *Being There: Putting Brain*, *Body*, *and World Together Again* (1997). Clark introduces Umwelt as a conceptual precursor to “niche-dependent sensing” in robotics and defines it as “the set of environmental features to which a given type of animal is sensitized” (Clark, 1997, p. 24). His explanation of the concept is short and centers on a citation of Uexküll’s popular passage on the tick. From Clark’s description, it is not clear whether an *Umwelt* involves experience or not, but he embeds it in a larger account of a robot called Herbert and concludes that the “similarity between the operational worlds of Herbert and the tick is striking” (25). This makes it at least possible to understand *Umwelt* as unconcerned with experience from Clark’s account, but whether other authors who suggest that robots have *Umwelten* were directly influenced by Clark or not cannot be established. Clark himself, at least, clearly believes that *Umwelt* without experience is possible, more than two decades after Being There: “A simple robot could […] properly be assigned an Umwelt. But the simple affordance-sensitive robot need not thereby experience any world at all” (Clark, 2019, p. 284). This is in contrast to Uexküll, for whom having an *Umwelt* is the same as experiencing it.

## *Umwelt* as a Bridge Between Ecological Psychology and Enactivism

Baggs and Chemero (2018, p. 5) argue that *Umwelt* allows ecological psychology to better account for the specific way in which each animal perceives its environment, bringing it closer to enactivism. In keeping with central tenets of the ecological approach, they argue that “the environment is not a separate mental realm,” but rather a mere “subset of the physical world, considered from the vantage point of an animal.” Their goal is then to show how *Umwelt* can be derived from an account of the physical world. They advance two complementary arguments. The first focuses on the environment and describes a single world which is continuous across the different scales of physical universe, species habitat, and individual *Umwelt*. The second focuses on the individual organism and explains how different physiological abilities as well as learned skills and acquired knowledge determine which affordances present in a given habitat become part of an individual’s *Umwelt*. I will focus here on the first argument and argue that it does not adequately account for experience because it treats the creation of *Umwelt* as selection, rather than construction, and thus does not address type 2 *Umwelt*. This latter sense of *Umwelt*, however, is what Baggs and Chemero need to get at if they want Uexküll’s thought to provide ecological psychology with an account of experience that is closer to the enactivist perspective.

The “key to Gibson’s theory of affordances” is his distinction between physical space and the environment of animals (Baggs and Chemero, 2018, p. 4). A central part of this concerns scale: the “physical world exists at all spatial and temporal scales, from nanoseconds and nanometers to millennia and galaxies” (Baggs and Chemero, 2018, p. 4). In contrast, the environment of animals occupies the “middle scale,” and for humans the “spatial scale of the environment is from millimeters to kilometers; the temporal scale is from hundreds of milliseconds to decades” (Baggs and Chemero, 2018, p. 4). This distinction gives us the impression of zooming in on the *Umwelt*, from the complete picture of the universe to just the tiny section of it that is relevant for a species, and even further to just the *Umwelt* of a given individual organism. This visualization is in concord with the attempt to describe one single continuous world, and matches the description of *Umwelt* as a “subset of the physical world” (Baggs and Chemero, 2018, p. 5). There is, however, one problem: “Most crucially, the physical world is inherently meaningless, but the environment is not; the environment contains affordances” (Baggs and Chemero, 2018, p. 5). If the physical world is a set which contains no meaning, no subset of it can contain any meaning either, by virtue of how sets and subsets are defined. Similarly, optical magnification can only enhance features which are already present in a visual phenomenon; we cannot zoom in on something that is not already there to begin with.

The central difficulty is that ecological psychology holds that there is just one world, one physical space, while Uexküll believes that “[s]pace as we think of it is the space with which the physicist deals, while intuited space as we look at it is the space of the biologist. The two are fundamentally different from one another” (von Uexküll, 1926, p. 42). Already committed to a single world, Baggs and Chemero can only select different parts of it and thus cannot move from type 1 *Umwelt* to type 2, i.e., they cannot account for the subjective experience of the organism. This is because the fundamental perspective of looking at the organism from the outside never changes, it merely zooms in on a subset of the environment in which this organism lives and on that subset of all affordances potentially available to the species which this specific individual can actually perceive and act upon, based on its history and physiology. In order to account for subjective experience, we have to consider both the scientific perspective on an organism from the outside and its own experience from the inside. Even in this account of an ecological psychology which deals with individual organisms and their type 1 *Umwelt*, this perspective from the inside, type 2 *Umwelt*, is left out.

## *Umwelt* in Enactivism

It may be that enactivism is better poised to grapple with *Umwelt* type 2 because it has had a strong focus on the experience of cognizing subjects from the very outset. Since *The Embodied Mind* (1991), a central influence on enactivism has been the structured exploration of one’s own consciousness, drawn predominantly from Eastern traditions and from the phenomenology of Edmund Husserl and Maurice Merleau-Ponty (Varela et al., 1991). In Thompson’s (2007) *Mind in Life* (2007), *Umwelt* appears at crucial points:

“This idea of a sensorimotor world—a body-oriented world of perception and action—is none other than von Uexküll’s original notion of an *Umwelt*. An *Umwelt* is an animal’s environment in the sense of its lived, phenomenal world, the world as it presents itself to that animal thanks to its sensorimotor repertoire” (Thompson, 2007, p. 59).

Thompson emphasizes the experiential character of *Umwelt*, which makes sense given that this reference to Uexküll follows a discussion of Maurice Merleau-Ponty and Kurt Goldstein, both of whom developed critical readings of Uexküll that grappled with the central role of experience (Merleau-Ponty, 1968, 1988; Goldstein, 1995). The process of constructing an *Umwelt* is explained as sense-making, an activity that each organism has to engage in constantly to maintain itself within the delicate bounds of its “needful freedom” (Jonas, 2001, p. 80; Thompson, 2007, pp. 146, 147). The needful freedom of the organism is what gives valence to the “[p]hysical and chemical phenomena” in the environment of the organism which, “in and of themselves, have no particular significance or meaning” (Thompson, 2007, pp. 153, 154). “Sense-making changes the physicochemical world into an environment of significance and valence, creating an *Umwelt* for the system” (Thompson, 2007, p. 147). In his exposition of Varela’s claim that “living is sense-making,” Thompson posits seven points, among them that “*[e]mergence of a self entails emergence of a world*. The emergence of a self is also by necessity the co-emergence of a domain of interactions proper to that self, an environment or *Umwelt*” (Thompson, 2007, p. 158).

Thompson’s account emphasizes central aspects of type 2 *Umwelt*, its experiential character, its emergence from the activity of every living organism, and the one-to-one mapping of subjects—or selves—to *Umwelten*. However, large parts of Uexküll’s thought have not been taken over into Thompson’s enactivist account: the entire semiotic vocabulary is left out. Where Uexküll uses signs to describe relationships of meaning, Thompson instead employs a notion of information that is derived in part from the work of Scott Kelso:

“What could be more meaningful to an organism than information that specifies the coordinative relations among its parts or between itself and the environment? This view turns the mind-matter, information-dynamics interaction on its head. Instead of treating dynamics as ordinary physics and information as a symbolic code acting in the way that a program relates to a computer, dynamics is cast in terms that are semantically meaningful” (Kelso, 1995, p. 145, quoted in Thompson, 2007, p. 58).

Thompson’s combination of type 2 *Umwelt* with Kelso’s notion of information is ingenious and important for two reasons: First, it shows that the essential insight of type 2 *Umwelt* can be retained while discarding much of Uexküll’s often cumbersome and idiosyncratic terminology. Second, Kelso’s work on dynamics and the notion of information that is part of it lie at the heart of 21st century ecological psychology. Its central role in both schools suggests that Kelso’s notion of information might be one good place from which to work toward a unified ecological-enactive approach as “a working metatheory for the study of minds” (Chemero, 2009; Baggs and Chemero, 2018).

## From Constructivism to Holism: Uexküll’s Musical Theory of Meaning

Fultot and Turvey (2019) reject Uexküll’s claim that each organism constructs its own world as representationalist. They highlight how the construction of *Umwelt* as modeled—in an idiosyncratic way—on Kantian epistemology parallels key aspects of cognitivism that ecological psychology rejects. Their rejection of *Umwelt* thus follows from their full appreciation of type 2 *Umwelt* and the process of its construction. Instead of the concept of *Umwelt* which entails that there are “as many worlds as there are subjects,” Fultot and Turvey develop an understanding of nature as a unified world in which all elements are harmoniously interconnected by melodies, harmonies, and counterpoints of meaning from a reading of Uexküll’s *A Theory of Meaning* (von Uexküll, 1926, p. 70, 2010).

To argue against Uexküll’s doctrine of many worlds, Fultot and Turvey recapitulate Gibson’s rejection of the Gestalt theorists’ subjectivist conception of *“[A]ufforderungscharakter”*, which Gibson translated as “affordance” (Fultot and Turvey, 2019, p. 14). They note the links between Gestalt theory, Gibson and Uexküll, but also emphasize the conceptual tensions between them. In his development of affordances as “organism-relative without being organism-dependent,” they take Gibson to be implicitly “targeting von Uexküll’s theory and theories like it” (15). The argument hinges on the question of whether there is “a unique, private access of each individual organism to its surroundings,” which Uexküll endorsed and Gibson rejected (15). Fultot and Turvey follow Gibson in tracing this view back to the fact that “no two individuals can occupy the same geographical point at the same time” (15). According to Gibson, the primary reason to think that each living organism lives in its own subjective world is based in “a narrow conception of optics and a mistaken theory of visual perception” (Gibson, 1979, p. 38, quoted in Fultot and Turvey, 2019, p. 15).

Read as a criticism of Uexküll, this appears to miss the mark. The problem that Uexküll’s constructivist account of *Umwelt* seeks to solve is posed in terms opposed to those of Gibson’s ecological approach. Where Gibson considered a single environment containing meaningful affordances and which “all inhabitants have an equal opportunity to explore,” for Uexküll the main explanatory work has to start earlier (Gibson, 1979, p. 38, quoted in Fultot and Turvey, 2019, p. 15). The problem is not that different organisms cannot occupy the same point in an environment at once, it is that they each have to construct their environments from scratch. Once an organism perceives meaningful affordances, indeed as soon as it experiences any environment at all, we are already *in medias res*, and much of what Uexküll describes has to have taken place already as the condition of the possibility of this experience. The *Umwelt* of an animal has to be accounted for because the colors that a bee sees are neither part of the objective material world described by the physicists, for whom there are “only waves, after all, and nothing more,” nor do they coincide with the colors that humans see von Uexküll (2010, p. 134). More than that, each bee has to generate their own experience as an organismic activity in contact with its physical surroundings. The dynamical relationship of the organism to its physical surroundings gives rise to its subjective experience of its *Umwelt*.

Fultot and Turvey point out the sharp contrast between Uexküll’s Kantian constructivism and the conceptual underpinnings of Gibson’s direct realism. As an alternative to the notion of *Umwelt*, they introduce a second theory of meaning in nature found in Uexküll’s *Bedeutungslehre* (first published in 1940). This later text, which was published in English translation as *A Theory of Meaning* together with the slightly earlier *Foray*, develops an account of why the structures of living organisms fit so perfectly into their environment of other organisms and the inorganic world. As a staunch critic of Darwinism, Uexküll sees the harmonious composition of the natural world as evidence of a greater plan that orders the realm of the living into one overarching symphony of meaning, composed of countless melodies, harmonies, and counterpoints.

Fultot and Turvey highlight a series of parallels between Uexküll’s musical theory of meaning and Gibson’s emphasis on a “complementarity between organism and environment” that enables the former to directly pick up on affordances specified by information available in the latter (18). They outline two ways of conceiving the organism/environment relationship, as the familiar representationalist dualism that is to be rejected, or as a *duality*, which involves a different kind of symmetry between organism and environment. Where a representational symmetry involves “the preservation of all the relations and their order,” duality preserves “the number of relations but can transform their quality and revert their order” (19). Representation entails the creation of duplicates or copies, while duality works on the basis of correspondences, such as the peg of a cogwheel fitting into the socket of another.

Two problems with this evaluation of Uexküll’s musical theory of meaning arise: First, the account of meaning in nature as one great holistic symphony does not replace the constructivism of *Umwelt*, it complements it. Second, the arguments that Uexküll provides in support of this musical theory of meaning are quite different from the Kantian constructivism of *Umwelt*, but they are not free from conceptual baggage. On the contrary, they are drawn in part from Hans Driesch’s neovitalism and Goethe’s romantic holism. These views depart so radically from generally accepted philosophical assumptions in contemporary philosophy of the natural sciences that they require substantial amounts of conceptual work before they can be integrated into existing accounts of embodied cognition.

In their careful reading of *A Theory of Meaning*, Fultot and Turvey identify an “implied realism about the properties of the environment” (20). They cite Uexküll’s description of an octopus, where he states that the “incompressibility of the water is the precondition for the construction of a muscular swimming sac” (von Uexküll, 2010, p. 173). The incompressibility of the water does depend on the existence of the octopus as subject, illustrating the point that the role played by seawater in the meaningful activity of swimming is “organism-independent yet organism-*relative”* (Fultot and Turvey: 20). “Meaning is *already there*, so to speak” (Fultot and Turvey: 20). Two points relativize this realism. Even though we are taking an external perspective on the octopus that allows us to understand its place in a system of meaning by reference to a larger harmonious whole rather than purely as constructed by the octopus itself, *A Theory of Meaning* does not constitute a departure from Uexküll’s *Umwelt* theory. Besides the musical theory of meaning, the text still contains the same constructivist view of how a subject creates its *Umwelt:* “The sun is a light in the sky. The sky is, however, a product of the eye, which construct here its farthest plane, which includes all of environmental space” (von Uexküll, 2010, p. 190). According to Uexküll, this principle of how a subject constructs its phenomenal *Umwelt* is valid for octopi just as it holds for humans, and scientists too can only ever investigate their own *Umwelten* (von Uexküll, 2010, p. 207). The incompressibility of the water has octopus-independent reality, but always within the *Umwelt* of a subject. In this case, the subject is a musical ecologist analyzing “the *octopus as subject* in relation to the *seawater as carrier of meaning”* (von Uexküll, 2010, p. 173). Uexküll ends both *A Theory of Meaning* and the *Foray* with the reminder that the limitations of *Umwelt* also apply to our scientific endeavors. Fultot and Turvey are clearly exaggerating when they consider “his Kantian views having been abandoned” (24).

As *Umwelt* grows out of Uexküll’s reading of Kant, so his musical theory of meaning grows out of romanticist holism and the neovitalism of Hans Driesch. Uexküll and Driesch had met in Naples in the 1890s, where Uexküll was researching the physiology of *Eledone moschata* while Driesch studied the development of sea urchins (Mildenberger and Herrmann, 2014a, p. 5, 2014b, pp. 274–276). Driesch demonstrated that “a sea urchin germ cell cut in half became not two half, but two whole sea urchins of half the size,” which to Uexküll demonstrated that nature is not exhausted by mechanical explanation and warranted far-reaching conclusions: “Everything physical can be cut with a knife—but not a melody” (von Uexküll, 2010, p. 194). Uexküll agrees with Karl von Baer that there is a “goal-pursuing quality in the emergence of living beings” and identifies musical harmony as the driving force of this teleological embryogenesis:

“planned embryonic development […] beings with the three beats of a simple melody: morula, blastula, and gastrula. Then, as we know, the development of the buds of the organs begins, which is fixed in advance for every animal species. This proves to us that the sequence of formal development has a musical score which, if not sensorily recognizable, still determines the world of the senses. This score also controls the spatial and temporal extension of its cell material, just as it controls its properties” (von Uexküll, 2010, pp. 159, 160).

To today’s reader, the role of the melody in this account of embryogenesis is at best a poetic placeholder that has to be replaced by scientific explanations and at worst a kind of vital life force. The latter option is unfortunately very plausible, as Driesch was a leading proponent of neovitalism (Mildenberger and Herrmann, 2014a, pp. 5, 6). While Fultot and Turvey choose to ignore Uexküll’s appeals to an overarching plan in nature as “creationist-sounding,” it seems that his vitalistic tendencies are tightly linked to the musical account of biological form and cannot so easily be separated from it (Fultot and Turvey, 2019, p. 18).

The second philosophical source from which Uexküll’s musical theory of meaning draws its strength is a romanticist holism. The melodies, harmonies, and counterpoints are an *explanation* rather than a *description* of the organization of the biological realm only if we accept a holistic worldview in which wholes determine their parts in accordance to an overarching and preexisting schema, rule, or “*primal image [Urbild]*” (von Uexküll, 2010, p. 159). This principle, which we saw in action in the embryogenic formation of a sea urchin in accordance to its “primal score,” goes back to Goethe’s famous *Urpflanze* (160). Frederick Amrine has outlined Uexküll’s deep debt to Goethe, with whom he sides against Newton in the question of color perception (Amrine, 2015, p. 50). Amrine explains Uexküll’s musical theory of meaning as an instance of Goethean ecology, and his arguments are convincing: A central refrain in *A Theory of Meaning* is developed from Goethe:

“If the flower were not bee-like,
If the bee were not flower-like,
The harmony would never succeed.” (von Uexküll, 2010, p. 198)

This is based in Goethe’s claim that:

“Were the eye not sunlike
It could never gaze upon the sun” (von Uexküll, 2010, p. 190).

The vision of nature that Uexküll expresses in musical terms is deeply grounded in romanticist holism, and the “meaning plan” which guarantees that its parts, developing in accordance to their “primal images” and “primal melodies,” fit into the overarching harmony is guaranteed by Goethe’s spinozist “God-Nature” (von Uexküll, 2010, p. 192). The problem for us today is that Uexküll is tapping into an entirely different conception of science than the one that has dominated the last centuries and that is accepted today. For Uexküll, “[m]eaning is the pole star by which biology must orient itself, not the impoverished rules of causality” (von Uexküll, 2010, p. 160). Besides its roots in romanticism, Di Paolo provides another good reason for caution about holistic harmony:

“while we must avoid the flattening out of the biological and psychological worlds into a series of mechanisms, we must also be cautious with the theme of the harmony of the world […] if we understand harmony as a primordial state of mutually counterpunctual relations of meaning (“the spider is fly-like”). Here, what is excluded, to repeat, are the precarious conditions and the ongoing, effortful processes by which meaning is achieved whatever the timescale, whether evolutionary, developmental, or behavioral” (Di Paolo, 2019, p. 254).

In the context of Uexküll’s holism, it is worth mentioning that his belief in a great whole that unifies individual organisms under one rule of meaning found a deeply disturbing expression in his *Staatsbiologie*. In 1920, Uexküll first published this interpretation of the state as a biological organism. After Germany’s defeat in the First World War, Uexküll had become increasingly antisemitic and began channeling this conviction in his academic writing (Mildenberger and Herrmann, 2014b, pp. 294, 295). A second edition of the book published in 1933 included a partially rewritten section on “the diseases of the state [Die Krankheiten des Staates],” which identifies members of foreign races who are detrimental to the state as “parasites” (von Uexküll, 1933, pp. 59, 72). Uexküll concludes the book by praising the “ingenious doctor” into whose care Germany has delivered itself as a “deeply sick patient”—a reference to Adolf Hitler (von Uexküll, 1933, p. 79; Winthrop-Young, 2010, pp. 226, 227). These connections between romanticist holism and fascism in Uexküll’s work are deeply disconcerting (cf. Harrington, 1996). To be clear, the musical holism of *A Theory of Meaning* itself contains none of these vile totalitarian biologisms, and using its concepts in ecological psychology would not thereby import anything objectionable. However, it still seems important to mention this aspect of Uexküll’s work in any discussion of his holism.

One problem with using *A Theory of Meaning* to bolster an account of the organism/environment relation in embodied cognition is that its main function in Uexküll’s work is to provide an account of the appearance of design in nature. This was a desideratum for Uexküll because he firmly rejected the Darwinian account. That is not a pressing question for us today. Few people doubt that our best explanation for why the spider spins a web that corresponds so well to the structure of the fly will invoke Darwinian evolution. The potentially useful part of *A Theory of Meaning* is its account of meaning understood through the musical concepts of harmony, melody, counterpoint, and so on. However, most of the arguments given in support of this musical account derive from neovitalism and Goethean ecology. Were we to remove all elements that are not immediately compatible with our contemporary understanding of natural science, *A Theory of Meaning* does not seem to offer much argumentative support for an account of how organisms are attuned to their environments. There are interesting parallels to Gibson’s ecological approach, and some of Uexküll’s descriptions provide vivid illustrations to the principles of ecological psychology, but it is unclear what is added to its explanatory power or conceptual clarity by bringing in Uexküll’s musical idiom.

Even if we accept Uexküll’s musical theory of meaning and the overarching harmony guaranteed by its elusive plan, this only accounts for the appearance of design in nature, not for the experience of living subjects. What the musical holism explains is why the different organisms observed in nature appear to fit each other and their environments so perfectly. This explains why the *Merkwelt* and *Wirkwelt* of type 1 *Umwelt* fit together in functional cycles. It does not contain an account of why the execution of functional cycles involves subjective experience. Uexküll’s musical holism does not appear to offer any help in grappling with the problem of accounting for subjective experience in a scientifically grounded account of mind—the “explanatory gap between consciousness and nature” (Thompson, 2007, p. 10).

## Conclusion

We have seen that Uexküll’s concept of *Umwelt* is fundamentally concerned with subjective experience. A deflated account of *Umwelt* as a mere ‘engineering concept’ is still widespread in the philosophy of cognitive science, but it does not help address the problem of subjective experience and is only tangentially related to Uexküll’s philosophical project. Since the point of contention between the ecological and enactive approaches is the status of subjective experience, only the full sense of *Umwelt* as the unique subjective phenomenal world of each organism is relevant for this debate. However, this sense of Umwelt is not immediately compatible with deeply held commitments of ecological psychology, as Fultot and Turvey point out. If some specifics of Uexküll’s Kantian constructivism are omitted, *Umwelt* seems compatible with enactivism, but this compatibility depends on the degree to which enactivism is understood as constructivist.

Uexküll’s compositional theory of meaning, which Fultot and Turvey propose to adopt instead of *Umwelt*, poses some difficulties that have been pointed out above. It does not do the same job as *Umwelt*, since it does not account for subjective experience as such but only for the observable complementarity between the different parts of nature. Its original purpose, to provide an alternative explanation for the appearance of design in nature for those who reject Darwinism, does not seem useful to us today. Importantly, the compositional theory of meaning is based entirely in Uexküll’s adoption of Goethe’s romanticist philosophy of nature and Hans Driesch’s neovitalism. This means that it does not come with less metaphysical baggage than *Umwelt*, just with a different kind. If ecological psychology were to adopt the compositional theory of meaning, it would have to deal explicitly with this philosophy of nature that appears to be quite far removed from the philosophical foundations of the ecological approach.

To establish a common philosophical foundation for a joint ecological-enactive approach to the study of cognition, more work seems necessary than importing new concepts from Uexküll, or from some other thinker. The main benefit of *Umwelt* for this particular debate may be that it provides a structured and principled account of how subjective experience constitutes the worlds we perceive and act in which allows ecological psychologists and enactivists to systematically assess their points of agreement and rejection. By itself, this will not unite the two approaches. But may well give us a clearer picture of the specific structure of their disagreements and the underlying philosophical commitments that cause them. The difficulty of accounting for subjective experience in any theory of mind suggests that a true unification of the two approaches may only be possible if both sides are at least in principle willing to question some of their most longstanding beliefs. If the central philosophical intuition of *Umwelt* is taken to be correct, mind after Uexküll can only be understood in light of the foundational character of subjective experience.

## Author Contributions

The author confirms being the sole contributor of this work and has approved it for publication.

## Conflict of Interest

The authors declare that the research was conducted in the absence of any commercial or financial relationships that could be construed as a potential conflict of interest.

## Footnotes

1 My gratitude belongs to the two reviewers who have provided numerous helpful and critical suggestions to all parts of this analysis. My forays into the reception of Uexküll’s thought have benefitted greatly from the works of Carlo Brentari and Frederick Amrine, to whom I am likewise grateful.

2 Uexküll’s appeals to Kant concern almost exclusively the role that the structure of the subject plays in enabling its own experience of time and space. Interestingly, Uexküll makes no mention of the *Critique of Judgment* and Kant’s notion of organisms as autonomous *Naturzwecke* (natural purposes), even though this aspect of Kant’s thought has been argued to play a crucial role in the work of early biologists like Karl von Baer, whom Uuexküll cites favorably and who was “one of the principal architects of the teleo-mechanist research program” according to Timothy Lenoir’s account of the influence of Kant on early 19th century German biology (Lenoir, 1982, p. 16). While some teleo-mechanists treated organismic teleology more as a methodological commitment, Uexküll’s use of romanticism instead of Kant’s *Critique of Judgment* leads him to treat it as a fully real part of nature.

3 For a discussion of this distinction in enactivism and ecological psychology, see Fultot et al. (2016).

4 The references to Kant’s *Critique of Pure Reason* in this paper reflect the level of Uexküll’s reception thereof, rather than the intricacies of Kant’s actual philosophical system. A careful comparison between the two would be worthwhile, but constitutes a separate research project. Langthaler (1992) provides some short but useful remarks on the matter.
